# Pharmacy-based dispensing of antimicrobial agents without prescription in India: appropriateness and cost burden in the private sector

**DOI:** 10.1186/s13756-015-0098-8

**Published:** 2015-12-11

**Authors:** Anita Shet, Suba Sundaresan, Birger C. Forsberg

**Affiliations:** Department of Pediatrics, St. John’s Medical College Hospital, Bangalore, India; Department of Public Health Sciences, Karolinska Institutet, Stockholm, Sweden; Department of Pediatrics, Division of Infectious Diseases, St. John’s Research Institute, Sarjapur Road, Bangalore, 560034 India

**Keywords:** Antimicrobial use, Antimicrobial resistance, Non-prescription dispensing, Pharmacy practice

## Abstract

**Background:**

Inappropriate antibiotic use for treatment of common self-limiting infections is a major problem worldwide. We conducted this study to determine prevalence of non-prescription sale of antimicrobial drugs by pharmacies in Bangalore, India, and to assess their associated avoidable cost within the Indian private healthcare sector.

**Methods:**

Between 2013 and 2014, two researchers visited 261 pharmacies with simulated clinical scenarios; upper respiratory tract infection in an adult and acute gastroenteritis in a child. Using a pre-defined algorithm, the researchers recorded questions asked by the pharmacist, details of medicines dispensed, and instructions regarding drug allergies, dose and side effects.

**Results:**

Antimicrobial drugs were obtained without prescription from 174 of 261 (66.7 %) pharmacies visited. Instructions regarding dose of these drugs were given by only 58.0 % pharmacies. Only 18.4 % (16/87) of non-antimicrobial-dispensing pharmacies cited the need for a prescription by a medical practitioner. None gave advice on potential side effects or possible drug allergies. In the upper respiratory infection simulation, 82 (71.3 %) of the 115 pharmacies approached dispensed antimicrobials without a prescription. The most common antimicrobial drug prescribed was amoxicillin (51.2 %), followed by azithromycin and ciprofloxacin (12.2 % each). Among 146 pharmacies where acute gastroenteritis was simulated, 92 (63.0 %) dispensed antimicrobials. Common ones were fluoroquinolones (66.3 %), particularly norfloxacin in combination with metronidazole. Standard treatment for diarrhea such as oral rehydration solution and zinc was prescribed by only 18 of 146 (12.3 %) pharmacies. Assuming the average cost of a 5-day course of common antimicrobials in India is $1.93, with 2.5 and 2.1 annual episodes of adult upper respiratory and childhood gastrointestinal infections respectively, and with 30–45 % of the population of 1.3 billion visiting pharmacies, the estimated cost of unnecessary antimicrobial drugs dispensed by pharmacies in India would range from $1.1 to 1.7 billion.

**Conclusions:**

The study shows that dispensing of antimicrobial drugs without prescription by pharmacies in the private sector in India within an urban setting was unacceptably high, thus placing a high burden on healthcare expenditure. There is an urgent need to institute measures to curb unnecessary antimicrobial usage in India, address market incentives and involve pharmacists as partners for creating awareness among communities.

**Electronic supplementary material:**

The online version of this article (doi:10.1186/s13756-015-0098-8) contains supplementary material, which is available to authorized users.

## Background

Antimicrobial agents have transformed the health of the world and have saved millions of lives worldwide. Notwithstanding the phenomenal success of antimicrobials in improving quality of life globally, the dangers of overuse of antimicrobials are becoming apparent. Non-prescription-based inappropriate antimicrobial use for treatment of common self-limiting infections is a major problem worldwide, with implications that range from development of antimicrobial resistance, contribution towards increasing medical costs and increased drug-associated adverse effects [1].

India carries a very high burden of infectious diseases and consequently has one of the highest antimicrobial usages in the world [2]. In the wake of the emergence of highly resistant strains like the New Delhi metallo-betalactamase-1 superbug, the government of India formulated a National Policy for Containment of Antimicrobial Resistance in 2011 [3]. Anecdotal evidence suggests that inappropriate prescribing practices by pharmacies and drug stores is rampant in India despite the presence of governmental policies and rigorous campaigning by national bodies like the Indian Academy of Pediatrics [4]. Personnel in charge of most pharmacies and drug stores are predominantly businessmen rather than professionally trained healthcare providers [5]. It is common practice in many Indian community pharmacies to dispense antibiotics on demand from the patient or customer even though a valid prescription from a registered medical provider is not available [6]. However there is a dearth of systematic studies that quantify the magnitude of the problem. We hypothesized that there will be significant dispensing of unwarranted antimicrobial drugs, which would add a substantial cost burden to society. In this study, we used a set of simulated symptoms of common viral illnesses and documented the prevalence of non-prescription sales of antimicrobial agents by pharmacies in a metropolitan city in southern India.

## Methods

### Study site

The study site was Bangalore, the capital of Karnataka, a state in southern India. According to the 2011 census, Bangalore has a population of 9.6 million with 11 % children under 5 years of age. It has a population density of 4381 per square kilometer, and is one of the fastest growing cities in India with a growth rate of 47 % over the past decade.

### Study design and sampling

We conducted a cross-sectional study observational study in 2013–2014. Based on previous surveys, we estimated a sample size of approximately 250 for a prevalence of non-prescription-based antimicrobial sales of 50 %, a 95 % confidence level and a 5 % margin of error [5, 7]. Commercial licensed pharmacies and drug stores in the municipal areas of Bangalore were mapped and listed based on their address. These pharmacies were each assigned a number using a computerized random number generator, and 10 % of these were selected for inclusion in the study using a random sampling method. This study was approved by the Institutional Ethical Committee of St. John’s Medical College Hospital, Bangalore (Reference No: 171/2011).

### Study tools

Two researchers posing as community customers approached the selected drug stores and simulated two clinical scenarios. A pre-defined validated algorithm for the dialogue between the researcher and the pharmacist was used. The two case scenarios simulated were that of an upper respiratory tract infection in an adult and acute gastroenteritis in a child (Additional files 1 and 2). The researcher simulating the upper respiratory infection presented with complaints of cough, nasal discharge and fever since four days. In the case of acute gastroenteritis the ‘patient’ was the researcher’s afebrile four-year-old child with complaints of loose stools for two days. After the simulated cases were presented the researchers noted if the pharmacist asked any further questions about the condition. Two levels of demand were explored; the first level included the request for a ‘medicine’ to alleviate the described symptoms. The medicine given by the pharmacist was noted. If no antimicrobial agent was dispensed, the researchers adopted the second level where they specifically asked for a ‘stronger’ medicine. Details of all medicines dispensed were recorded and, in case of antimicrobials, additional instructions by the pharmacist regarding dose, duration and side effects were noted. Queries from the pharmacist regarding possible drug allergies, and non-pharmacological advice for symptom alleviation, including referral to a medical practitioner were also noted.

### Statistical analysis

Descriptive statistical analyses of the results were performed. Chi square tests were used to compare categorical variables and Student *t* test was used to compare two mean values. All analyses were performed using Stata v13.

## Results

### General findings

Within the Bangalore urban area, 2640 commercial licensed pharmacies and drug stores were identified. Among the 10 % randomly selected 264 pharmacies, three were found to have closed, and hence a total of 261 pharmacies were included in the study. Using a computerized random number generator, these were randomly distributed into the “upper respiratory infection” group (*n* = 115) and the “acute gastroenteritis” group (*n* = 146). Among the 261 pharmacies included in the study, 84.2 % were pharmacy-only stores, while the remaining were pharmacy-general stores that also functioned as drug stores. Antimicrobial drugs were obtained without a valid prescription in 66.7 % (174/261) of pharmacies visited. Among these, 55.6 % (145/261) pharmacies dispensed antimicrobials at the first level of demand, and the remaining dispensed antimicrobials when a ‘stronger’ medicine was requested. None of the pharmacies provided counseling on expected side effects, nor queried about potential drug allergies. Instructions regarding dose and duration of the antimicrobial drugs were given by 58.0 % (101/174) and 51.1 % (89/174) respectively. Only 18.4 % (16/87) of pharmacies that did not prescribe antimicrobials stated the need for a prescription by a medical practitioner. No differences were observed in the sales of antimicrobials between pharmacy-only stores and pharmacy-general stores (*p* = 0.2).

### Upper respiratory tract infection simulation

Antimicrobial drugs were obtained without a prescription from 71.3 % (82/115) of pharmacies in which upper respiratory tract infection symptoms were presented (Table 1). At the first level of demand, 53.9 % (62/115) pharmacies gave antimicrobials, and the remaining 20 pharmacies acquiesced when a request for a ‘stronger’ medicine was made. No queries on drug allergies were made. Among dispensed antimicrobials, the most common was amoxicillin (51.2 %) followed by azithromycin and ciprofloxacin (12.2 % each) (Table 2). None of the pharmacies warned the patient of the expected side effects of the drugs. Only 21.7 % (25/115) asked the patient to visit the doctor, while 18.3 % (21/115) advised non-pharmacological treatment like steam inhalation (Table 1).

**Table 1:** Actions and advice from pharmacies in the upper respiratory tract infection and acute gastro-enteritis simulations

Type of action and advice	Proportion of pharmacies in each simulated condition	
	Upper respiratory tract infection in adult	Acute gastro-enteritis in child
	*n* = 115 (%)	*n* = 146 (%)
Dispensation of antibiotics without prescription	82 (71.3)	92 (63.0)
Queries regarding drug allergies	Nil	Nil
Instruction on dose of dispensed antibiotic(s)	79 (96.3)	22 (23.9)
Instruction on duration of dispensed antibiotic(s)	75 (91.5)	14 (15.2)
Instruction on side effects of dispensed antibiotic(s)	Nil	Nil
Need for prescription (cited by non-dispensing pharmacies)	3/33 (9.1)	13/54 (24.1)
Instruction on need to visit doctor	25 (21.7)	49 (33.6)
Non-pharmacological advice	Steam inhalation: 21 (18.3)	ORS and/or zinc: 18 (12.3)

ORS: Oral rehydration solution

**Table 2:** Proportion of antibiotic types that were dispensed without a valid prescription by pharmacies sampled in the study

Antimicrobial agents dispensed	Proportion of pharmacies dispensing antibiotics in each simulated condition	
	Upper respiratory tract infection in adult	Acute gastro-enteritis in child
	*n* = 115 (%)	*n* = 146 (%)
Amoxicillin	42 (51.2)	-
Amoxicillin-clavulanate	2 (2.4)	-
Ampicillin-cloxacillin	4 (4.9)	-
Cephalexin	2 (2.4)	-
Cefixime	2 (2.4)	-
Azithromycin	10 (12.2)	-
Roxithromycin	3 (3.7)	-
Ciprofloxacin	10 (12.2)	-
Levofloxacin	3 (3.7)	-
Ofloxacin (alone)	4 (4.9)	7 (7.6)
Norflaxacin (alone)	-	8 (8.7)
Norfloxacin + Metronidazole	-	38 (41.3)
Ofloxacin + Metronidazole	-	9 (9.8)
Ofloxacin + Ornidazole	-	7 (7.6)
Metronidazole (alone)	-	14 (15.2)
Furazolidone	-	9 (9.8)

### Acute gastroenteritis simulation

Among the 146 pharmacies that were presented with the scenario of the child with acute gastroenteritis, 92 (63.0 %) dispensed antimicrobials without a prescription (Table 1). At the first level of demand, 56.8 % (83/146) were given antimicrobials. Only a quarter of the pharmacies asked for more clinical details. Fluoroquinolones (66.3 %) were the most common group of antimicrobials dispensed (Table 2). Norfloxacin in combination with metronidazole was the most common ‘combination agent’ dispensed by 41.3 % of the pharmacies. Instructions regarding dose and duration of the drugs were given by 23.9 % and 15.2 % respectively, and none of the pharmacy personnel explained about the expected side effects. Of the 54 pharmacies that did not give an antimicrobial, 13 (24.1 %) stated that they could only dispense antimicrobials with a prescription. Oral rehydration solution with or without zinc was given by only 12.3 % (18/146) of the pharmacies, and 33.6 % (49/146) of the pharmacy personnel advised a visit to the doctor (Table 1).

### Cost considerations

The excess financial burden of consuming unnecessary antimicrobials can be estimated by basing our calculations on prior epidemiological data; the average cost of a five-day course of common antibiotics in India was estimated at $1.93 [8]; the average number of minor upper respiratory infections in adults as 2.5 episodes per person annually [9], and number of childhood gastrointestinal infections as 2.1 diarrhea episodes per child per year [10, 11] (Table 3). For a family of 4 (2 adults and 2 children) the cost of unnecessary antimicrobials alone would amount to 1.4 % of per capita income. The population of 1.3 billion in India [12], relies predominantly on private rather than public healthcare facilities; World Bank figures indicate that the proportion of out-of-pocket health expenditure in India in 2008–09 was about 85 % of total health expenditure [13]. Health utilization studies in India indicate that over 90 % of the population attends private healthcare sites [14], although about 40–50 % of these may be facilities that dispense complementary and alternative medicine [15]. Thus about 30–45 % of the population with minor illnesses is likely to visit allopathic pharmacies for purchasing their medications. Extrapolating our study findings that 66 % of pharmacies inappropriately dispense antimicrobials, the estimated cost of unnecessary antimicrobial drugs dispensed by pharmacies in India would be in the range of $1.1 to 1.7 billion.

**Table 3:** Cost estimation of the practice of non-prescription-based antimicrobial sales in the private sector

	Scenario 1	Scenario 2
Total population in India (in 2013) [13]	1267	1267
Population 15 years and above [13]	900	900
Population 0–14 years [13]	367	367
Cost of antibiotics - 5-day course [9]	$ 1.9	$ 1.9
Number of annual upper respiratory infection episodes in adults [10]	2.5	2.5
Number of annual diarrhea episodes in children [11,12]	2.1	2.1
Total number of upper respiratory infection episodes in adults	2249	2249
Total number of diarrhea episodes children	772	772
Total number of ALL episodes in the population	3021	3,021
Proportion of ill population visiting allopathic pharmacies [15,16]	30 %	45 %
Total number of ill population visiting pharmacies	906	1359
66 % dispense antimicrobial drugs (based on study)	598	897
Total annual cost of unwarranted antimicrobial drugs	1136	1704

The estimation takes into account existing data on population size, infection episodes per year for each age group, and proportion of population visiting allopathic pharmacies. Two scenarios are considered based on whether 30 % or 45 % of the ill population visits pharmacies. Numbers shown are in millions

## Discussion

Antimicrobial drugs without prescription can be obtained with ease from over two-thirds of the randomly sampled pharmacies and drug stores in our city. Our study results indicate that antimicrobial dispensation takes place with scant consideration for possible adverse effects and drug interactions, and the dose and duration or both are frequently not addressed. The choice of antimicrobial is often inappropriate, and the use of irrational combination antimicrobials is rampant. Such an amalgam of potent factors can predispose profoundly to adverse public health issues such as increasing community antimicrobial resistance and escalating costs of healthcare. The urban healthcare market in India is characterized by a supply of drugs through a plethora of small businesses and private providers of health services; these drug shops and pharmacies are commonly found in all Indian communities down to town level where common practices prevail [16]. Owing to the similarities in market practice with reference to drug sales and prescribing practices, our findings may be applicable to other Indian urban and semi-urban areas.

Antimicrobial agents remain one of the most commonly purchased drugs globally. A systematic review showed that non-prescription use occurred worldwide and accounted for 19–100 % of antimicrobial use in countries other than those in northern Europe and North America [17]. The sale of antimicrobials is largely unregulated, without involvement of a licensed trained pharmacist, and is often without prescription [17]. Drug stores and pharmacies in India and other parts of Asia are fast becoming substitutes for primary healthcare providers in urban areas [18]. Studies from other parts of the world with poor regulation of drug-dispensing policies show variable rates of non-prescription antibiotic sales. A Greek study in 2001 showed that 71 % of pharmacies agreed to sell broad-spectrum antibiotics to patients with low-grade fever and rhinosinusitis [19]. Several other simulation studies conducted in Spain and Brazil have shown that antimicrobial agents could be easily obtained in spite of regulations prohibiting such practice [20, 21]. In Tanzania, oral antibiotics were given to 81 % and 95 % of those who presented with diarrhea and upper respiratory infection respectively [22]. Other Indian studies particularly indicate alarming prevalence of inappropriate antibiotic consumption [5, 23, 24]. Our study used simulated illnesses that were most likely viral in etiology, and therefore showed that antimicrobials obtained without prescription were often not appropriately dispensed. Other assessments have indicated dispensation at sub-optimal doses and durations [5, 25]. A study of non-prescription-based sales of antibiotics by pharmacists from Thailand discovered that the closer the drug stores were to major hospitals, the more appropriate was the nature of the dispensing from those stores [26]. Widespread consumption of antimicrobials is the main force driving selection of pressure resulting in antimicrobial resistance. Communities with frequent use of antimicrobials without prescription commonly also have high community antimicrobial resistance rates [27, 28]. A study in Spain correlated the pattern of antimicrobial resistance with the community antibiotic consumption trends and found a decline in resistance with decrease in the use of antibiotics in the community [29].

What determines antimicrobial prescribing and dispensing practices among healthcare workers, pharmacists and community members in low-and-middle income countries (LMICs)? A comprehensive review indicated that factors include lack of knowledge of healthcare workers, public beliefs, demand from patients, expensive or poorly available diagnostic tests, economic influences and marketing pressures [30]. Qualitative studies from India with focus group discussion among pharmacists, doctors and the public reveal similar factors including the pressure to sell antimicrobials nearing their expiry date [7, 23]. In a survey done in Nagpur India, majority of pharmacists reported that their prime source of continuing education was from sales representatives from pharmaceutical companies [5]. A focus group discussion study among retail pharmacists in Delhi showed that commercial interests constitute a major force in driving sales of inappropriate antibiotics [23]. A community-based survey from Mongolia revealed that self-medication of antibiotics was common, particularly for childhood illnesses, and was driven by widespread misperceptions that antibiotics can ‘cure’ viral illnesses [31]. The authors concluded that one of the reasons for antibiotic misuse may be its widespread availability and poor oversight by community pharmacies [31]. Market financial incentives where antibiotics may be more profitable than more rational treatment for diarrhea such as zinc or oral rehydration solution may be another reason for widespread antibiotic dispensing [32].

In India, antimicrobials command the largest share in the Indian pharmaceutical market (18 %) [33]. We have estimated that the community’s financial burden pertaining to unnecessary antimicrobials dispensed by pharmacies in India can be over $ 1 billion. Overuse of antimicrobials represents a considerable financial burden among the less economically advanced section of the society. Equally, inappropriate use through suboptimal dosing and poor adherence can pose a tremendous cost liability from an individual and public health standpoint. In an attempt to curb the rising threat posed by the irrational sale of antimicrobials, in 2011 the Government of India amended the Drugs and Cosmetics Rule of 1945 to include certain drugs under a new Schedule H1. Currently, 46 drugs are enlisted under this category of which 35 are antimicrobial agents. For any drug under this category to be dispensed, a valid prescription is mandatory. In addition the pharmacist will have to maintain a sales record in a register with additional details like name and contact of the prescribing doctor and the quantity of the drug dispensed. This amendment may have limited efficacy in curbing indiscriminate use of antimicrobials as drugs like beta-lactam agents, older fluoroquinolones including ciprofloxacin and ofloxacin, and macrolides such as azithromycin that are most likely to be dispensed without prescription, are not included under schedule H1 [34]. In addition, studies across the globe have shown that state regulations may not be sufficient to curb the practice of inappropriate antimicrobial dispensing [35]. Although regulating sale of antimicrobials is essential to promote appropriate use, it should be only a part of a more comprehensive strategy. Other important measures include increasing awareness of rational antimicrobial use and antimicrobial resistance, and involving pharmacists as partners for creating this awareness among communities. Reports from some LMICs suggest that multi-component interventions can be successful in reducing non-prescription sale of antimicrobials. In Chile, strict legislations with simultaneous public health campaigns had an immediate and significant impact on sale of antimicrobials, while little or no effect was seen in other places such as Mexico and Venezuela where enforcement was not combined with public awareness campaigns [36]. Interventions that include different degrees of regulation and education have been shown to be effective in reducing inappropriate antibiotic dispensing by drug sellers in Vietnam and Thailand [25, 37].

Regulation of antimicrobial sales in resource-limited countries should be balanced with the prevailing healthcare burdens. A major cause of child mortality in these countries is inadequately treated infections, particularly serious respiratory infections [38]. Antimicrobials have been life saving on a broad scale, and non-prescription use may have certainly enabled wide access in these settings. A prime challenge is ensuring that truly deserving populations receive the antimicrobials they need, while at the same time restricting rampant non-prescription antimicrobial use. Achieving this balance is the task of interdisciplinary cooperative forces from governments, scientific advisory committees, public health academicians, economists and activists from many of the concerned nations.

Our study did not distinguish whether the dispensing workforce in pharmacies were licensed and trained personnel. The study was also limited by the fact that simulated cases were presented to pharmacies, rather than actual case scenarios, although this procedure helped maintain uniformity in our data collection. Since the sample size was limited, and only pharmacies from one urban area in India were included, our study may be limited by non-inclusion of other practices in rural areas, where alternative systems of medicine may be more popular. This diversity of practices was however, considered during the costing analysis. Notwithstanding these limitations, our findings bring to light an important public health problem that need to be addressed with a multi-disciplinary approach. Possible solutions to address this complex conundrum include training of pharmacists and surveillance of antimicrobial drug dispensation on a nationwide scale. Improved regulation of antimicrobial dispensing practices, coupled with appropriate market financial incentives can also help curtail inappropriate antimicrobial dispensing practices.

## Conclusions

Our study revealed unacceptably high rates of non-prescription antimicrobial use in India that includes inappropriate dispensing of antimicrobial drugs that adds an inordinately high cost burden to society. Syntheses of our findings along with data from other countries suggest that these practices are remarkably similar in other parts of the world. Concerted interventions that incorporate a multi-pronged approach can minimize non-prescription antimicrobial dispensing practices.

## Additional files

Additional file 1:
**URTI simulation questionnaire, 2013.** (PDF 70 kb) Additional file 2:
**Child diarrhea questionnaire, 2013.** (PDF 83 kb)

## Abbreviation

LMIC: Low-and-middle income countries
