# Supplementary material for: Pharmacy-based dispensing of antimicrobial agents without prescription in India: appropriateness and cost burden in the private sector
Source: Antimicrob Resist Infect Control. 2015 Dec 11;4:55. doi: 10.1186/s13756-015-0098-8 (PMC4676185; doi:10.1186/s13756-015-0098-8)
Supplement: Additional file 1: — URTI simulation questionnaire, 2013. (PDF 70 kb) [file 13756_2015_98_MOESM1_ESM.pdf]

URTI simulation questionnaire

**Date:**	**Time:**
**Pharmacy Name and Address (inc Phone)**	**Pharmacy No: (from listing)**
	**Language used:**
	**Research assistant:**

You have a cough, running nose and fever since a few days. You go to the pharmacy and ask for treatment for this.		
**Section 1 (Questions***)* *Wait for the 'pharmacist' to respond now. If he asks you any of the following questions, these should be your answers. If no questions asked, mark N in the last column and skip to section 2.*		
Question	***Your answer***	***Pharmacist asked question (Y/N)***
How long since you have the cold/cough/ fever (circle which symptom he asked)?	4 days	
Is the fever high?	I haven't measured, just know I am feverish	
Any headache / body ache?	Occasionally	
Any sputum?	Yes	
List any other questions he asks you and your response.		
**Section 2 treatment.** *If no antibiotics are prescribed, ask the pharmacist the following: Can you give me a strong medicine for this. I have a lot of work to finish in the next 2 days.*<br>*If cough syrup is given, record details below and at the end of the interaction say you will buy it later.*<br>*If antibiotics are prescribed, ask if it is ok that you just buy today's dose for now and buy only that much. Record the details below (indicate antibiotic.*		
Name of drug	***Particulars he tells you (Y/N***	***Details of particulars (NA if not told)***
1.	Dosage<br>Duration<br>Side effects	
2.	Dosage<br>Duration<br>Side effects	
Prescribes antibiotics	*Y /N*	*(if Y fill particulars below, else skip to section 3)*
Name of antibiotic	***Particulars he tells you (Y/N)***	***Details of particulars (NA if not told)***
1.	Dosage<br>Duration<br>Side effects	
2.	Dosage<br>Duration<br>Side effects	
**Section 3 (Advice).** *List non pharmacological advice if any*		
Advice	Y/N	
Steam inhalation		
Visit a doctor		
Other (list)		

URTI simulation questionnaire

**Date:**	**Time:**
**Pharmacy Name and Address (inc Phone)**	**Pharmacy No: (from listing)**
	**Language used:**
	**Research assistant:**

You have a cough, running nose and fever since a few days. You go to the pharmacy and ask for treatment for this.		
**Section 1 (Questions*)*** *Wait for the 'pharmacist' to respond now. If he asks you any of the following questions, these should be your answers. If no questions asked, mark N in the last column and skip to section 2.*		
Question	***Your answer***	***Pharmacist asked question (Y/N)***
How long since you have the cold/cough/ fever (circle which symptom he asked)?	4 days	
Is the fever high?	I haven't measured, just know I am feverish	
Any headache / body ache?	Occasionally	
Any sputum?	Yes	
List any other questions he asks you and your response.		
**Section 2 treatment.** *If no antibiotics are prescribed, ask the pharmacist the following: Can you give me a strong medicine for this. I have a lot of work to finish in the next 2 days.*<br>*If cough syrup is given, record details below and at the end of the interaction say you will buy it later.*<br>*If antibiotics are prescribed, ask if it is ok that you just buy today's dose for now and buy only that much. Record the details below (indicate antibiotic.*		
Name of drug	***Particulars he tells you (Y/N***	***Details of particulars (NA if not told)***
1.	Dosage<br>Duration<br>Side effects	
2.	Dosage<br>Duration<br>Side effects	
Prescribes antibiotics	*Y /N*	*(if Y fill particulars below, else skip to section 3)*
Name of antibiotic	***Particulars he tells you (Y/N)***	***Details of particulars (NA if not told)***
1.	Dosage<br>Duration<br>Side effects	
2.	Dosage<br>Duration<br>Side effects	
**Section 3 (Advice).** *List non pharmacological advice if any*		
Advice	Y/N	
Steam inhalation		
Visit a doctor		
Other (list)		
