# Supplementary material for: Pharmacy-based dispensing of antimicrobial agents without prescription in India: appropriateness and cost burden in the private sector
Source: Antimicrob Resist Infect Control. 2015 Dec 11;4:55. doi: 10.1186/s13756-015-0098-8 (PMC4676185; doi:10.1186/s13756-015-0098-8)
Supplement: Additional file 2: — Child diarrhea questionnaire, 2013. (PDF 83 kb) [file 13756_2015_98_MOESM2_ESM.pdf]

.

Child Diarrhea simulation questionnaire

**Date:**	**Time:**
**Pharmacy Name and Address (inc Phone)**	**Pharmacy No: (from listing)**
	**Language used:**
	**Research assistant:**
Your child (boy - 4 years) has loose motions since two days. You go to the pharmacy and ask for treatment for this. Don't say anything else at this point.	
**Section 1 (Questions)** *Wait for the 'pharmacist' to respond now. If he asks you any of the following questions, these should be your answers. If no questions asked, mark N in the last column and skip to section 2.*	

Question	***Your answer***	***Pharmacist asked question (Y/N)***
How many stools a day?	Say 5-6 times a day	
Any fever?	I haven't measured	
Any pain in abdomen or colic?	Mild	
Child eating/drinking/loss of appetite?	Child a bit tired, not eating so much because he feels unwell	
Colour of stool ?	Yellow	
Blood / mucus in stool?	Not noticed	
List any other questions he asks you and your response below		
Do you have a prescription?	No	
Go and see a doctor?	I do not have so much time. Also don't want to spend	

**Section 2: Treatment**		
**Name of drug**	**Particulars he tells you (Y/N**	**Details of particulars (NA if not told)**
1.	Dosage<br>Duration<br>Side effects	
2.	Dosage<br>Duration<br>Side effects	

If antibiotics are prescribed, say that you will return with money and buy the medicines. Or, ask if it is ok that you just buy today's dose for now and buy only that much. If antibiotics and anti motility are prescribed skip to section 3
If antibiotics are not prescribed, ask the pharmacist the following: "Can you give me a strong medicine for this. I have a lot of work to finish in the next 2 days"

Prescribes antibiotics	Y /N	(if Y fill particulars below, else skip to section 3)
**Name of antibiotic**	**Particulars he tells you(Y/N)**	**Details of particulars (NA if not told)**
1.	Dosage<br>Duration<br>Side effects	
2.	Dosage<br>Duration<br>Side effects	

Say that you will return with money and buy the medicines.

Section 3 (Advice). List non pharmacological advice if any

**Advice**	**Y/N**	
ORS / salt sugar water / rice water / coconut water /		
Visit a doctor		
Zinc syrup		
Other (list)		

Section 4 (Any other comment)
